# Intersection of Acquired and Congenital Cardiovascular Disorders in Pregnancy∗

**DOI:** 10.1016/j.jacadv.2022.100042

**Published:** 2022-05-25

**Authors:** Kathryn J. Lindley

**Affiliations:** Cardiovascular Division, Washington University School of Medicine, St. Louis, Missouri, USA

**Keywords:** congenital heart disease, hypertension, preeclampsia, pregnancy

**Figure:**
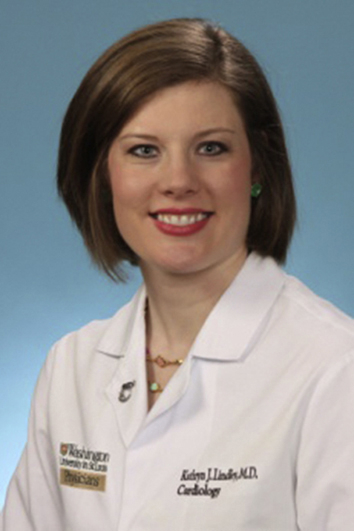

Maternal mortality has been steadily rising in the United States over the last several decades, with cardiovascular disease as the leading cause of maternal deaths.^1^ Though the majority of these deaths are related to acquired cardiovascular conditions, the number of pregnant women with congenital heart disease (CHD) continues to increase in the setting of improved pediatric surgical techniques.^2^ Unfortunately, it is known that up to 60% of patients with CHD have a significant lapse in CHD care during their transition from pediatric to adult CHD clinics, with pregnancy being one of the leading reasons for return to care.^3^ This has important implications for preconception counseling and cardiovascular prevention, which may impact pregnancy outcomes.

In this issue of *JACC: Advances*, Goldstein et al^4^ evaluate risk factors and outcomes associated with hypertensive disorders of pregnancy (HDPs) in maternal congenital heart disease, through a retrospective cohort analysis of the longitudinal Alberta Pregnancy-Birth cohort. HDPs are an increasingly prevalent complication of pregnancy with serious maternal and fetal consequences.^5^ Not only are women at risk of immediate cardiovascular complications of pregnancy including heart failure (HF), stroke, and myocardial infarction when experiencing HDPs, HDP are clearly and consistently associated with an increased risk of long-term cardiovascular complications including HF, stroke, ischemic heart disease, and chronic hypertension (HTN).^6^^,^^7^ Thus, identifying risk factors and potentially actionable targets for improving HDP outcomes among high-risk women is essential.

In their analysis, Goldstein et al compared maternal and fetal outcomes, stratified into 4 groups based on the presence or absence of CHD and HDP: +CHD/-HDP, +CHD/+HDP, -CHD/-HDP, -CHD/+HDP. Notably, HDPs were significantly more common among women with CHD (11.2% vs 8.1%, *P* < 0.0001), with both gestational HTN and preeclampsia/eclampsia occurring more commonly in the CHD group. Further, the HDP among women in the CHD group was more severe, as evidenced by early-onset preeclampsia/eclampsia (diagnosed <34 weeks gestation).

Importantly, the key drivers for the increased incidence of HDP among women with CHD were primarily acquired risk factors, namely chronic HTN and diabetes mellitus, with adjusted odds ratios of 4.56 and 3.33, respectively, among women with CHD. Both chronic HTN and diabetes mellitus were significantly more prevalent among the CHD cohort, despite being overall slightly younger than the control group. This truly highlights the importance of preconception and interpregnancy preventive care for the CHD population—with weight management, cardiometabolic screening, and dietary and exercise modifications—essential both for pregnancy health as well as prevention of long-term acquired cardiovascular disease.^7^, ^8^, ^9^, ^10^

The only CHD-specific condition that was associated with HDPs in this study was coarctation of the aorta. CHD disease complexity and other specific CHD lesions were not associated with HDPs, though a true effect may not have been identified due to coding errors which were biased toward simple lesions, and small numbers of complex defects. The findings of increased risk of HDPs in women with coarctation of the aorta is consistent with previous smaller cohort studies and are plausible due to the high prevalence of underlying chronic HTN, as well as persistent endothelial dysfunction and potential for placental hypoperfusion among women with repaired or unrepaired coarctation of the aorta.^11^^,^^12^ Given the known benefit of prophylactic low-dose aspirin in reducing the risk of preeclampsia among women with moderate or strong risk factors, these findings merit further study for confirmation and raise the question of whether this population may also potentially benefit from prenatal aspirin treatment.^13^

Short-term adverse maternal and fetal outcomes were increased for both women with CHD and those with HDP; however, HDPs conferred a greater risk of delivery hospitalization complications than did CHD. It is known that HDPs are a major driver of cardiovascular complications of pregnancy and in the early postpartum period, accounting for 7% of all maternal deaths in the United States, including 44% of deaths in the first week postpartum and 21% of deaths between weeks 1 and 6 after delivery.^14^^,^^15^ Further, HDPs are the leading cause of postpartum hospital readmissions—reducing both maternal and infant quality of life and driving health care costs.^16^

Women with both CHD and HDPs carried the highest risk for complicated delivery hospitalization outcomes and 1-year death or readmission, highlighting the need for closely monitoring women with CHD for the development of HDPs, as well as potential complications of HDPs such as HF. The postpartum period is the highest risk period for cardiovascular complications among women with pre-existing cardiovascular disease, and blood pressure is known to increase during this period among women with HDPs.^7^^,^^17^^,^^18^ Women with underlying CHD are known to be at an increased risk of postpartum HF symptoms,^19^ and HDPs are a known risk factor for peripartum HF.^7^^,^^18^^,^^20^ Thus, women with CHD who have pregnancies complicated by HDPs warrant close monitoring, especially in the early postpartum period to assess for symptoms of uncontrolled HTN and HF. This study further highlights the importance of adequate blood pressure control and achievement of euvolemia in women with underlying CHD prior to discharge to home, as well as early fourth trimester follow-up.^7^

The authors of this study should be congratulated on their work investigating a common but serious cardiovascular complication of pregnancy among a high-risk group of patients. The results of this study have implications across the reproductive life span of women with CHD—including early pregnancy and contraception counseling for adolescents, effective transition of care from pediatric to adult CHD clinics, preventive preconception and interpregnancy cardiovascular care, the potential role for prenatal preeclampsia prevention strategies, intensified third and fourth trimester monitoring and management, and longitudinal risk factor modification.

## Funding support and author disclosures

The author has reported that she has no relationships relevant to the contents of this paper to disclose.
